# Deactivation of the JNK Pathway by GSTP1 Is Essential to Maintain Sperm Functionality

**DOI:** 10.3389/fcell.2021.627140

**Published:** 2021-02-25

**Authors:** Marc Llavanera, Yentel Mateo-Otero, Ariadna Delgado-Bermúdez, Sandra Recuero, Samuel Olives, Isabel Barranco, Marc Yeste

**Affiliations:** ^1^Biotechnology of Animal and Human Reproduction (TechnoSperm), Institute of Food and Agricultural Technology, University of Girona, Girona, Spain; ^2^Unit of Cell Biology, Department of Biology, Faculty of Sciences, University of Girona, Girona, Spain

**Keywords:** ezatiostat, GSTP1-JNK heterocomplex, mitochondria, sperm functionality, mammalian sperm

## Abstract

Fifty percent of male subfertility diagnosis is idiopathic and is usually associated with genetic abnormalities or protein dysfunction, which are not detectable through the conventional spermiogram. Glutathione *S*-transferases (GSTs) are antioxidant enzymes essential for preserving sperm function and maintaining fertilizing ability. However, while the role of GSTP1 in cell signaling regulation via the inhibition of c-Jun N-terminal kinases (JNK) has been enlightened in somatic cells, it has never been investigated in mammalian spermatozoa. In this regard, a comprehensive approach through immunoblotting, immunofluorescence, computer-assisted sperm assessment (CASA), and flow cytometry analysis was used to characterize the molecular role of the GSTP1–JNK heterocomplex in sperm physiology, using the pig as a model. Immunological assessments confirmed the presence and localization of GSTP1 in sperm cells. The pharmacological dissociation of the GSTP1–JNK heterocomplex resulted in the activation of JNK, which led to a significant decrease in sperm viability, motility, mitochondrial activity, and plasma membrane stability, as well as to an increase of intracellular superoxides. No effects in intracellular calcium levels and acrosome membrane integrity were observed. In conclusion, the present work has demonstrated, for the first time, the essential role of GSTP1 in deactivating JNK, which is crucial to maintain sperm function and has also set the grounds to understand the relevance of the GSTP1–JNK heterocomplex for the regulation of mammalian sperm physiology.

## Introduction

In humans, about 30–50% of fertilizations fail because of male subfertility problems, usually related to abnormal sperm count, motility, and/or morphology (Ghuman and Ramalingam, 2017). However, over 50% of male subfertility diagnosis is of unknown etiology, since no abnormalities are detected in conventional semen analysis (sperm count, motility, and morphology) (Ghuman and Ramalingam, 2017). These patients are diagnosed as normozoospermic subfertile men (i.e., male idiopathic subfertility). Male idiopathic subfertility has been associated with genetic abnormalities (Carrell et al., 2006) and low levels of sperm-specific proteins (Parent et al., 1999; Bracke et al., 2018). While the general processes of sperm maturation, capacitation, and fertilization are well described, the underlying molecular mechanisms that take place in mammalian sperm cells remain mostly unknown (Klinovska et al., 2014). The origin of male idiopathic subfertility may be explained by molecular defects in these processes, since they are not detectable through the conventional spermiogram (Bracke et al., 2018). For this reason, characterization of signaling pathways and posttranslational modifications in mammalian sperm cells are of utmost interest for the andrology field.

Several studies reported the association between male idiopathic subfertility or infertility and some null genotypes of glutathione *S*-transferases (GSTs) (Aydos et al., 2009; Safarinejad et al., 2010; Vani et al., 2010; Tang et al., 2012; Kan et al., 2013; Lakpour et al., 2013; Song et al., 2013; Kolesnikova et al., 2017). Moreover, recent studies have evidenced the essential role of these antioxidant enzymes in sperm protection against oxidative stress and preservation of sperm function and fertilizing ability (Llavanera et al., 2019b, 2020). The first evidence of GST activity in mammalian sperm dates back to 1978 in murine species (Mukhtar et al., 1978), and the first report confirming the presence of the Pi class of GSTs (GSTP1) was published in 1998 in goat sperm (Gopalakrishnan et al., 1998). Since then, several proteomic profiling studies have identified GSTP1 in the sperm cells of a wide range of mammalian species, including humans (Wang et al., 2013), mice (Vicens et al., 2017), pigs (Pérez-Patino et al., 2019), cattle (Peddinti et al., 2008), and coatis (Rodrigues-Silva et al., 2018). In somatic cells, the main well-defined function of GSTP1 is cell signaling regulation via inhibition of the c-Jun N-terminal kinase (JNK)–C-Jun pathway (Adler et al., 1999; Wang et al., 2001; Turella et al., 2005). In non-stressed cells, GSTP1 is able to inhibit JNK kinase activity by blocking the JNK-binding site to C-Jun, forming a GSTP1–JNK heterocomplex. However, under cellular stress conditions, a GSTP1 aggregation followed by its dissociation from the heterocomplex leads to an increase in JNK activity (Adler et al., 1999; Wang et al., 2001; Turella et al., 2005). Recently, the JNK signaling cascade has been reported to be involved in sperm capacitation and apoptosis (Luna et al., 2017), which may undercover the role of GSTP1 in sperm physiology. However, the role of GSTP1 and the JNK–C-Jun pathway in mammalian sperm still remains unknown.

Ezatiostat or Terrapin 199 (TER) is a specific inhibitor of the GSTP1–JNK heterocomplex, used as an anticancer drug (Wu and Batist, 2013). After intracellular de-esterification, which is a process that commonly occurs in sperm cells (Griveau and Le Lannou, 1997), the active form of TER binds to GSTP1, blocking its JNK-binding site and, therefore, inhibiting the formation of the GSTP1–JNK heterocomplexes (Mathew et al., 2006). This inhibition enables JNK phosphorylation and activation of the subsequent pathway.

Along these lines, understanding the molecular role of the GSTP1–JNK heterocomplex in mammalian sperm physiology is much warranted. Herein, cell biology and immunological approaches were performed through pharmacologically inhibiting the formation of the GSTP1–JNK heterocomplex, prior to analyzing sperm quality and functionality parameters, the presence and localization of GSTP1, and the activation of JNK. Therefore, the present study aimed to investigate the function of this heterocomplex in mammalian sperm physiology, using the pig as a model, which has recently been stablished as a suitable animal model for research in human reproduction (Zigo et al., 2020). Accordingly, we hypothesized that the dissociation of the GSTP1–JNK heterocomplex, known to occur under cellular stress conditions, enhances the JNK signaling pathway and disrupts sperm physiology. The results obtained in this study can be used as a starting point for further investigations seeking the molecular basis of sperm dysfunction and may contribute to shedding light into the diagnosis of idiopathic male infertility.

## Materials and Methods

### Reagents

Chemicals and reagents were purchased from Sigma-Aldrich (Saint Louis, MO, United States), unless otherwise indicated. TER was reconstituted in dimethyl sulfoxide (DMSO) to a stock solution of 64 mM. Fluorochromes [SYBR-14, propidium iodide (PI), merocyanine 540 (M540), Yo-Pro-1, 5,5′,6,6′-tetrachloro-1,1′,3,3′-tetraethyl-benzimidazolylcarbocyanine iodide (JC1), Fluo3-AM (Fluo3), hydroethidine (HE), and fluorescein-conjugated peanut agglutinin/PI (PNA)] were purchased from Life Technologies (Thermo Fisher Scientific, Carlsbad, CA, United States). SYBR-14, M540, Yo-Pro-1, JC1, Fluo3, and HE were reconstituted in DMSO, whereas PI and PNA were diluted in phosphate-buffered saline (PBS) 1X. Antibody against GSTP1 (ref. MBS3209038) was purchased from MyBioSource (San Diego, CA, United States), whereas phospho-JNK (Thr183/Tyr185) antibody (pJNK, ref. 4668S) was purchased from Cell Signaling Technology (Danvers, MA, United States). Secondary anti-rabbit (ref. P0448) and anti-mouse (ref. P0260) antibodies conjugated with horseradish peroxidase for immunoblotting analysis were purchased from Dako (Derkman A/S, Denmark), whereas the secondary anti-rabbit antibody conjugated with Alexa Fluor 488 for immunofluorescence analysis was purchased from Thermo Fisher Scientific (ref. A32731).

### Animals and Ejaculates

Semen samples, commercially sold as pig artificial insemination (AI) seminal doses, were purchased from an authorized local AI center (Grup Gepork S.L., Masies de Roda, Spain) that followed ISO certification (ISO-9001:2008) and operates under commercial, standard conditions. Thirteen ejaculates (one ejaculate per boar, *n* = 13) from healthy and sexually mature Piétrain boars (1–3 years old) were collected using the gloved-hand method and diluted (33 × 10^6^ sperm/ml) using a commercial extender (Vitasem LD, Magapor S.L., Zaragoza, Spain). Packed ejaculates were transported at 17°C to the laboratory within 4 h after ejaculation. Since seminal doses were purchased from the aforementioned farm and the authors of this study did not manipulate any animal, no authorization from the institutional ethics committee was required.

### Experimental Design

All semen samples (*n* = 13) were split into three aliquots. The first aliquot was used to assess initial sperm quality and functionality (control-0h). The second and third aliquots were liquid-stored at 17°C for 72 h in the presence of (i) 100 μM ezatiostat (TER-72h) and (ii) the same volume of DMSO, as a vehicle control group (control-72h). Concentration of TER was selected based on the results obtained from a preliminary concentration test performed in our laboratory (Supplementary Figure 1), whereas storage time was decided following practical application criteria, considering that sows are artificially inseminated (two to three times per estrus) with AI doses stored until 72 h at 17°C. After 72 h, both groups were incubated at 38°C for 1 h prior to their analysis. All assessments were performed at every time point (control-0h, control-72h, and TER-72 h). Sperm motility, viability, plasma membrane stability, mitochondrial activity, intracellular calcium levels, intracellular superoxide levels, and acrosome membrane integrity were determined to evaluate sperm quality and functionality. The presence and localization of GSTP1 were explored by immunoblotting and immunofluorescence analyses, respectively. Finally, the activation of the JNK pathway was evaluated through immunoblotting analysis of JNK tyrosine and threonine phosphorylation. Raw data of sperm quality and functionality parameters of all treatments and time points are available as a data set (Supplementary Table 1).

### Sperm Motility Analysis

Sperm motility assessment was performed through a computer-assisted sperm analysis (CASA) system, using an Olympus BX41 microscope (Olympus, Tokyo, Japan) with a negative phase-contrast field (Olympus 10 × 0.30 PLAN objective, Olympus) connected to a personal computer containing the ISAS software (Integrated Sperm Analysis System V1.0, Proiser S.L., Valencia, Spain). Semen samples were incubated for 15 min at 38°C prior to motility assessment. Once incubated, 5 μl of each sample was examined in a prewarmed (38°C) Makler counting chamber (Sefi Medical Instruments, Haifa, Israel). Three technical replicates of at least 500 sperm per replicate were examined in each sample. Total motility (TMOT), progressive motility (PMOT), and average path velocity (VAP, μm/s) were used to evaluate sperm motility. A sperm cell was considered motile when VAP was ≥10 μm/s and progressively motile when the coefficient of straightness (STR) was ≥45%.

### Flow Cytometric Assessments

Sperm viability, plasma membrane stability, mitochondrial activity, intracellular calcium levels, intracellular superoxide levels, and acrosome membrane integrity assessments were conducted using a Cell Laboratory QuantaSC cytometer (Beckman Coulter, Fullerton, CA, United States) equipped with an argon-ion laser (488 nm) set at a power of 22 mW. Semen samples were diluted (2 × 10^6^ sperm/ml) in prewarmed PBS to a final volume of 600 μl prior to staining with the corresponding protocol. Sperm viability (SYBR-14/PI) (Garner and Johnson, 1995), plasma membrane stability (M540/Yo-Pro-1) (Rathi et al., 2001), mitochondrial membrane potential (MMP; JC1) (Ortega-Ferrusola et al., 2007), intracellular calcium levels (Fluo3/PI) (Harrison et al., 1993), intracellular superoxide levels (HE/Yo-Pro-1) (Guthrie and Welch, 2006), and acrosome membrane integrity (fluorescein-conjugated PNA/PI) (Nagy et al., 2003) were assessed. Extended flow cytometry protocols are described in Supplementary File 1.

The electronic volume (EV) gain, PMT voltages of optical filters (FL-1, FL-2, and FL-3), and fluorescence overlapping were set using unstained and single-stained samples of each fluorochrome. Flow rate, laser voltage, and sperm concentration were constant throughout the experiment. Sperm cells from debris events were distinguished using EV. Three technical replicates of at least 10,000 sperm per replicate were examined for each sample. As recommended by the International Society for Advancement of Cytometry (ISAC), Flowing Software (Ver. 2.5.1, University of Turku, Finland) was used to analyze flow cytometry data.

### Immunofluorescence Analysis

Semen samples were diluted in PBS (3 × 10^6^ sperm/ml) and fixed in 2% paraformaldehyde (Alfa Aesar, Haverhill, MA, United States) and washed twice. Two 150-μl aliquots of each sample were placed in an ethanol prerinsed slide and subsequently blocked and permeabilized for 40 min at room temperature (RT) with a blocking solution containing 0.25% (v:v) Triton X-100 and 3% (w:v) bovine serum albumin (BSA). Samples were incubated with anti-GSTP1 antibody (1:200, v:v) overnight, washed thrice, and subsequently incubated with an anti-rabbit antibody (1:400, v:v). In negative controls, the primary antibody was omitted. Then, 10 μl of Vectashield mounting medium containing 4,6-diamidino-2-phenylindole dihydrochloride (DAPI) was added prior to being covered and sealed with nail varnish. Finally, each sample was evaluated using a confocal laser scanning microscope (CLSM, Nikon A1R, Nikon Corp., Tokyo, Japan).

### Immunoblotting Analysis

Semen samples were centrifuged twice (3,000 × *g* for 5 min), and the sperm pellets were resuspended in lysis buffer (xTractor^TM^ buffer, Takara Bio, Kusatsu, Japan) following the manufacturer’s instructions. Then, samples were centrifuged (10,000 × *g* for 20 min at 4°C), and the supernatants were assessed for total protein quantification using a detergent-compatible method (Bio-Rad, Hercules, CA, United States). Finally, samples were stored at −80°C until analysis.

Twenty micrograms of total protein was diluted (1:1, v:v) in Laemmli reducing buffer 4X (Bio-Rad) and heated at 95°C for 7 min prior to being loaded onto a 12% polyacrylamide gel (Mini-PROTEAN^®^, TGX Stain-Free^TM^ Precast Gels, Bio-Rad) and electrophoresed for 2 h at 120 V. Total protein was visualized using a G:BOX Chemi XL system (Syngene, Frederick, MD, United States). Mini-PROTEAN^®^, TGX Stain-Free^TM^ Precast Gels contain a trihalo compound that allows fluorescent detection of tryptophan residues. Thereafter, proteins from the gel were transferred onto polyvinylidene difluoride (PVDF) membranes using the Trans-Blot^®^, Turbo^TM^ (Bio-Rad). Transferred membranes were blocked using 5% BSA and incubated with the anti-GSTP1 (1:5,000, v:v) or anti-pJNK (1:2,000, v:v) antibodies for 1 h in agitation at RT. Next, membranes were rinsed thrice and incubated with the secondary anti-rabbit antibody 1:10,000 (v:v) for GSTP1 and 1:4,000 (v:v) for pJNK. Then, membranes were washed five times, and bands were visualized through incubation with a chemiluminescent substrate (Immobilon^TM^ Western Detection Reagents, Millipore, United States) prior to scanning with G:BOX Chemi XL 1.4 (Syngene, India). Finally, membranes were stripped, and the process was repeated by replacing the primary antibody for the anti-α-tubulin antibody (1:100,000, v:v) and the secondary antibody for the anti-mouse antibody (1:150,000, v:v), as loading control and for normalization. In the pJNK assessment, Quantity One software package (Version 4.6.2, Bio-Rad) was used to quantify the bands of two technical replicates per sample, normalized using α-tubulin.

### Statistical Analysis

Plotting and statistical analysis of the results were performed using GraphPad Prism v.8 (GraphPad Software, La Jolla, CA, United States) and IBM SPSS for Windows v. 25.0 (IBM Corp., Armonk, NY, United States). Each biological replicate was considered a statistical case, and data were checked for normal distribution (Shapiro–Wilk test) and homogeneity of variances (Levene test). Sperm quality and functionality parameters, as well as normalized pJNK relative levels, were compared between treatments (control-0h, control-72h, and TER-72h) using a one-way ANOVA followed by Tukey’s multiple-comparison test. Data are shown as mean ± standard error of the mean (SEM). The level of significance was set at *p* ≤ 0.05.

## Results

### GSTP1 Is Present in Sperm Cells and Is Localized in the Principal and End Pieces of the Tail

**FIGURE 1:**
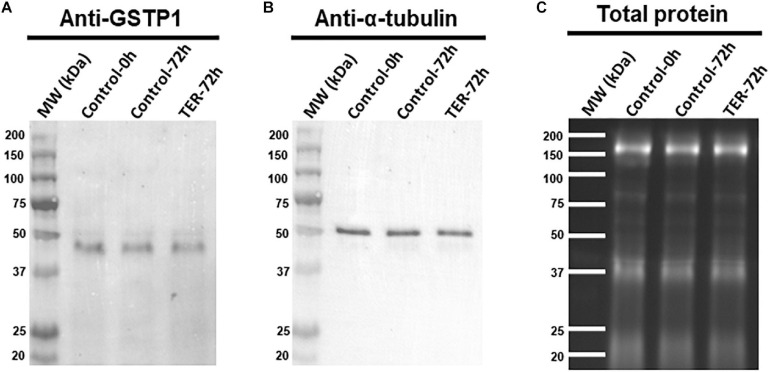
Immunoblotting analysis of GSTP1. Pig sperm lysates were incubated with **(A)** anti-GSTP1 antibody, **(B)** anti-α-tubulin antibody, and **(C)** total protein bands. Lanes: MW (kDa), molecular weight; Control-0h, semen samples at 0 h of storage; Control-72h, vehicle control (dimethyl sulfoxide; DMSO) semen samples at 72 h of storage at 17°C; TER-72h, semen samples treated with 100 μM ezatiostat (TER) for 72 h of storage at 17°C. Alpha-tubulin (anti-tubulin) and TGX Stain-Free^TM^ (total protein) were performed as complementary loading controls. These results are representative of three independent experiments (*n* = 3).

**FIGURE 2:**
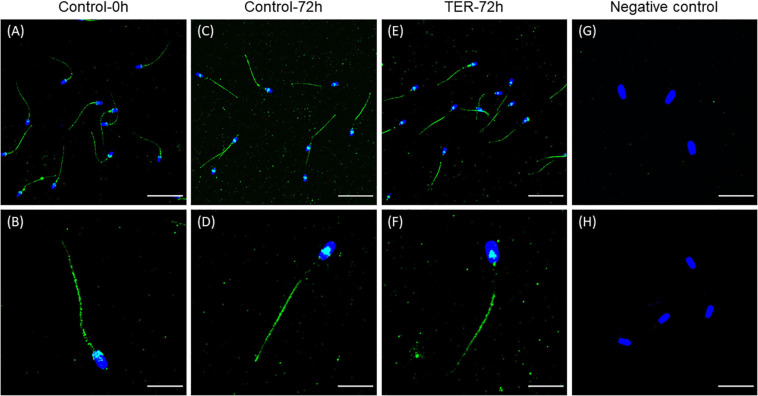
Immunolocalization of GSTP1 in pig sperm. **(A,B)** Control-0h, semen samples at 0 h of storage. **(C,D)** Control-72h, vehicle control (dimethyl sulfoxide; DMSO) semen samples at 72 h of storage at 17°C. **(E,F)** TER-72h, semen samples treated with 100 μM ezatiostat (TER) for 72 h of storage at 17°C. **(G,H)** Negative control. The nucleus is shown in blue (DAPI), whereas GSTP1 is shown in green. Scale bars: **(A,C,E)**: 30 μm; **(B,D,F)**: 15 μm; **(G)**: 20 μm; **(H)**: 25 μm. These results are representative of three independent experiments (*n* = 3).

The presence and localization of GSTP1 in sperm samples are presented in Figures 1, 2. Immunoblotting analysis of GSTP1 (Figure 1A) showed a single band of ∼48 kDa in all samples, whereas anti-α-tubulin (Figure 1B) showed a ∼50 kDa band. In Figure 2, a GSTP1 signal was observed in the posterior region of the head and the middle, principal, and end pieces of the tail of control-0h samples. In control-72h and TER-72h samples, the GSTP1 signal was observed only in the equatorial subdomain of the head and in the principal and end pieces of the tail.

### Inhibition of GSTP1–JNK Heterocomplex Formation by TER Induces Thr183 and Tyr185 Phosphorylation of JNK

**FIGURE 3:**
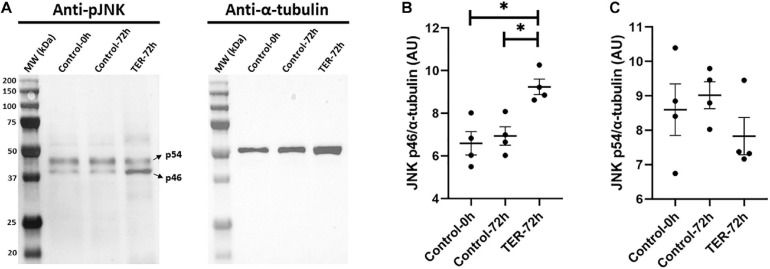
**(A)** Immunoblotting of pig sperm lysate for pThr183 and pTyr185 of JNK (p46 and p54 splicing variants) and α-tubulin. Subsequent band intensity quantification and normalization of **(B)** p46 and **(C)** p54 splicing variants of JNK, normalized using α-tubulin, showed a significant increase in Thr183 and Tyr185 phosphorylation in the p46 variant (*p* < 0.05), but not in the p54 variant (*p* > 0.05), in TER-treated samples (TER-72h) compared to control samples (control-0h and control-72h). Lanes: MW (kDa), molecular weight; control-0h, semen samples at 0 h of storage; control-72h, vehicle control (dimethyl sulfoxide; DMSO) semen samples at 72 h of storage at 17°C; TER-72h, semen samples treated with 100 μM ezatiostat (TER) for 72 h of storage at 17°C. These results are representative of four independent experiments (*n* = 4). *p ≤ 0.05.

Immunoblotting analysis of Thr183 and Tyr185 phosphorylation of JNK revealed a double-band pattern showing both p46 and p54 splicing variants of JNK (Figure 3). Anti-α-tubulin immunoblot showed a single band of ∼50 kDa, which corresponds to α-tubulin. Subsequent band quantification analysis of pJNK normalized using α-tubulin showed a significant increase (*p* < 0.05) in the relative levels of Thr183 and Tyr185 phosphorylation of the p46 splicing variant of JNK in TER-72h samples when compared to control-0h and control-72h samples. However, no effects of TER were observed in Thr183 and Tyr185 phosphorylation of the p54 splicing variant (*p* > 0.05).

### Sperm Viability Is Reduced by TER-Induced JNK Phosphorylation

**FIGURE 4:**
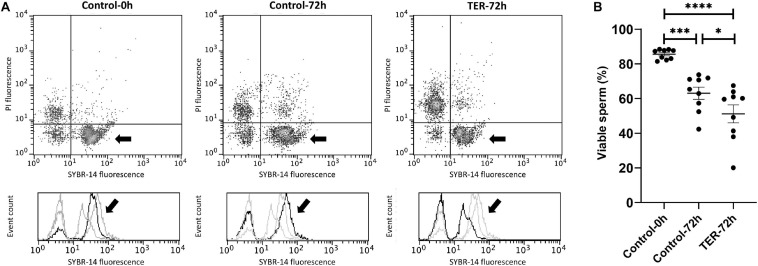
Flow cytometry analysis of sperm viability. **(A)** Representative flow cytometry dot plots of propidium iodide (PI) and SYBR-14 fluorescence and histograms showing the event count of SYBR-14 fluorescence intensity of all treatments. Black arrows show the flow cytometry population selected for analysis represented in **(B)**. Dark gray lines in histograms show the event count of its corresponding treatment, whereas light gray lines show the event count of the remaining treatments. **(B)** Mean, standard error of the mean (SEM), and sample distribution of the percentage of viable sperm in all treatments. Control-0h, semen samples at 0 h of storage; Control-72h, vehicle control (dimethyl sulfoxide; DMSO) semen samples at 72 h of storage at 17°C; TER-72h, semen samples treated with 100 μM ezatiostat (TER) for 72 h of storage at 17°C. Sample size (*n* = 9). *p ≤ 0.05; ***p ≤ 0.001; ****p ≤ 0.0001.

The percentage of viable sperm was higher (*p* < 0.05) in the semen samples of the control-0h group than in those of the TER-72h and control-72h groups (Figure 4). In addition, sperm viability was lower (*p* < 0.05) in the semen samples of the TER-72h group compared to those of the control-72h group.

### Phosphorylation of JNK by the Inhibition of GSTP1–JNK Binding Impairs Sperm Motility

**FIGURE 5:**
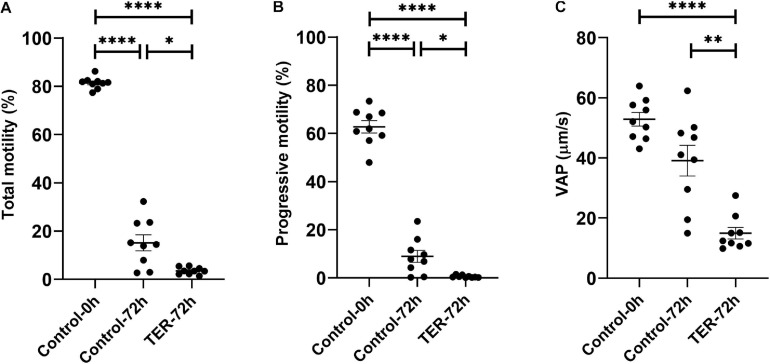
Computer-assisted sperm analysis (CASA) of pig semen samples. Mean, standard error of the mean (SEM), and sample distribution of the percentage of **(A)** total motile sperm, **(B)** progressively motile sperm, and **(C)** average path velocity sperm (VAP; μm/s) in all treatments. Control-0h, semen samples at 0 h of storage; Control-72h, vehicle control (dimethyl sulfoxide; DMSO) semen samples at 72 h of storage at 17°C; TER-72h, semen samples treated with 100 μM ezatiostat (TER) for 72 h of storage at 17°C. Sample size (*n* = 9). *p ≤ 0.05; **p ≤ 0.01; ****p ≤ 0.0001.

As shown in Figure 5, the percentage of total and progressive motile sperm was higher (*p* < 0.05) in the semen samples of the control-0h group than in those of the control-72h and TER-72h groups. However, VAP was lower (*p* < 0.05) in the semen samples of the TER-72h group compared to those of the control groups (0 and 72 h). Interestingly, total and progressive motility and VAP were significantly lower (*p* < 0.05) in the semen samples of the TER-72h group than in those of the control-72h group.

### Mitochondrial Activity Is Significantly Reduced by JNK Phosphorylation by the Inhibition of GSTP1–JNK Binding

**FIGURE 6:**
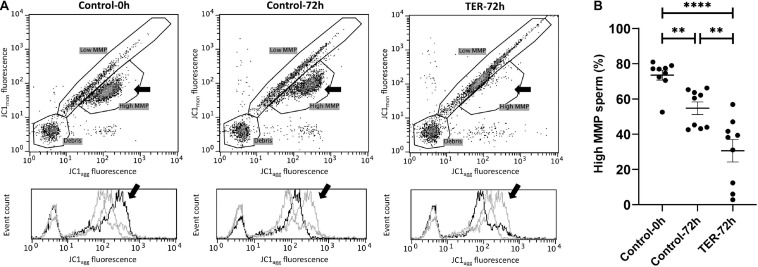
Flow cytometry analysis of pig sperm mitochondrial activity. **(A)** Representative flow cytometry dot plots of JC1 monomers (JC1_*mon*_) and JC1 aggregates (JC1_*agg*_) fluorescence and histograms showing the event count of JC1_*agg*_ fluorescence intensity of all treatments. Black arrows show the flow cytometry population selected for analysis represented in **(B)**. Dark gray lines in histograms show the event count of its corresponding treatment, whereas light gray lines show the event count of the remaining treatments. **(B)** Mean, standard error of the mean (SEM), and sample distribution of the percentage of high mitochondrial membrane potential (MMP) sperm in all treatments. Control-0h, semen samples at 0 h of storage; Control-72h, vehicle control (dimethyl sulfoxide; DMSO) semen samples at 72 h of storage at 17°C; TER-72h, semen samples treated with 100 μM ezatiostat (TER) for 72 h of storage at 17°C. Sample size (*n* = 9). **p ≤ 0.01; ****p ≤ 0.0001.

The assessment of MMP is presented in Figure 6. The percentage of sperm showing high MMP differed (*p* < 0.05) among the three groups, with the semen samples of the control-0h and TER-72h groups showing the highest and lowest percentages, respectively. Thus, a dramatic reduction in MMP was observed in the TER-72h group when compared to the control groups.

### Sperm Plasma Membrane Is Highly Destabilized by the Inhibition of GSTP1–JNK Binding and Subsequent JNK Phosphorylation

**FIGURE 7:**
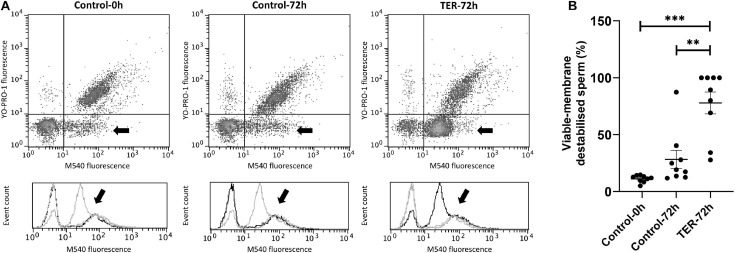
Flow cytometry analysis of plasma sperm membrane stability. **(A)** Representative flow cytometry dot plots of Yo-Pro-1 and merocyanine (M540) fluorescence and histograms showing the event count of M540 fluorescence intensity of all treatments. Black arrows show the flow cytometry population selected for analysis represented in **(B)**. Dark gray lines in histograms show the event count of its corresponding treatment, whereas light gray lines show the event count of the remaining treatments. **(B)** Mean, standard error of the mean (SEM), and sample distribution of the percentage of membrane-destabilized cells within the total viable sperm population in all treatments. Control-0h, semen samples at 0 h of storage; Control-72h, vehicle control (dimethyl sulfoxide; DMSO) semen samples at 72 h of storage at 17°C; TER-72h, semen samples treated with 100 μM ezatiostat (TER) for 72 h of storage at 17°C. Sample size (*n* = 9). **p ≤ 0.01; ***p ≤ 0.001.

As shown in Figure 7, sperm membrane stability was presented as the percentage of membrane-destabilized cells within the total viable sperm population. The percentage of viable sperm showing plasma membrane destabilization was higher (*p* < 0.05) in the semen samples of the TER-72h group than in those of the control groups (0 and 72 h). On the other hand, the plasma membrane stability of the semen samples did not differ between control groups (*p* > 0.05).

### Intracellular Superoxide Levels Were Increased by the Phosphorylation of JNK

**FIGURE 8:**
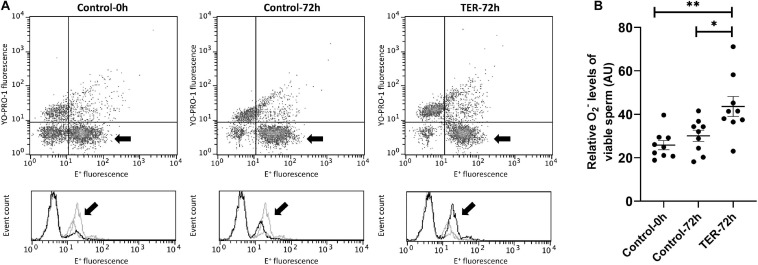
Flow cytometry analysis of intracellular superoxide levels. **(A)** Representative flow cytometry dot plots of Yo-Pro-1 and ethidine (E^+^) fluorescence and histograms showing the event count of E^+^ fluorescence intensity of all treatments. Black arrows show the flow cytometry population selected for analysis represented in **(B)**. Dark gray lines in histograms show the event count of its corresponding treatment, whereas light gray lines show the event count of the remaining treatments. **(B)** Mean, standard error of the mean (SEM), and sample distribution of the relative E^+^ fluorescence intensity of the viable sperm population in all treatments. Control-0h, semen samples at 0 h of storage; Control-72h, vehicle control (dimethyl sulfoxide; DMSO) semen samples at 72 h of storage at 17°C; TER-72h, semen samples treated with 100 μM ezatiostat (TER) for 72 h of storage at 17°C. Sample size (*n* = 9). *p ≤ 0.05; **p ≤ 0.01.

Figure 8 shows the relative E+ fluorescence intensity of the viable sperm population. No differences (*p* > 0.05) were observed in intracellular superoxide levels between semen samples of control groups (0 and 72 h). However, our results showed a significant increase (*p* < 0.05) in superoxide levels in semen samples of the TER-72h group compared to those of the control groups (0 and 72 h).

### Sperm Intracellular Calcium Levels and Acrosome Membrane Integrity Are Not Affected by TER-Induced JNK Phosphorylation

**FIGURE 9:**
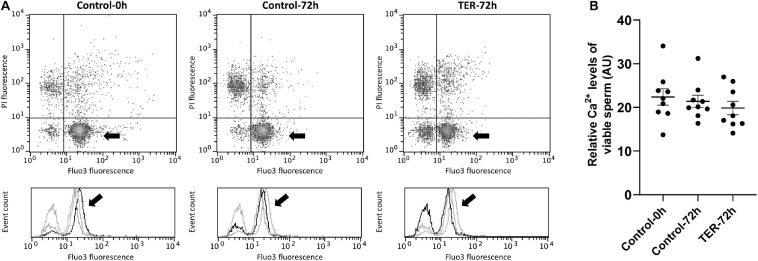
Flow cytometry analysis of intracellular calcium levels. **(A)** Representative flow cytometry dot plots of propidium iodide (PI) and Fluo3-AM (Fluo3) fluorescence and histograms showing the event count of Fluo3 fluorescence intensity of all treatments. Black arrows show the flow cytometry population selected for analysis represented in **(B)**. Dark gray lines in histograms show the event count of its corresponding treatment, whereas light gray lines show the event count of the remaining treatments. **(B)** Mean, standard error of the mean (SEM), and sample distribution of the relative Fluo3^+^ fluorescence intensity of the viable sperm population in all treatments. Control-0h, semen samples at 0 h of storage; Control-72h, vehicle control (dimethyl sulfoxide; DMSO) samples at 72 h of storage at 17°C; TER-72h, semen samples treated with 100 μM ezatiostat (TER) for 72 h of storage at 17°C. Sample size (*n* = 9). *p* > 0.05.

**FIGURE 10:**
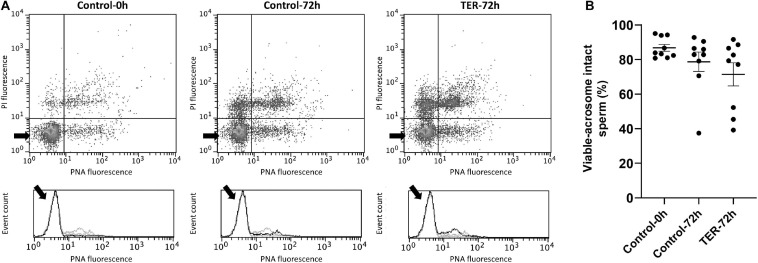
Flow cytometry analysis of acrosome integrity. **(A)** Representative flow cytometry dot plots of propidium iodide (PI) and fluorescein-conjugated peanut agglutinin/propidium iodide (PNA) fluorescence and histograms showing the event count of PNA fluorescence intensity of all treatments. Black arrows show the flow cytometry population selected for analysis represented in **(B)**. Dark gray lines in histograms show the event count of its corresponding treatment, whereas light gray lines show the event count of the remaining treatments. **(B)** Mean, standard error of the mean (SEM), and sample distribution of the percentage of viable sperm with an intact acrosome in all treatments. Control-0h, semen samples at 0 h of storage; Control-72h, vehicle control (dimethyl sulfoxide; DMSO) semen samples at 72 h of storage at 17°C; TER-72h, semen samples treated with 100 μM ezatiostat (TER) for 72 h of storage at 17°C. Sample size (*n* = 9). *p* > 0.05.

The relative Fluo3 fluorescence intensity (Fluo3^+^) of the viable sperm population (PI^–^) is presented in Figure 9, whereas the percentage of viable sperm with an intact acrosome (PNA^–^/PI^–^) is shown in Figure 10. No differences (*p* > 0.05) in intracellular calcium nor acrosome membrane integrity was observed among groups.

## Discussion

Several studies have evidenced the essential role of GSTs as molecular regulators of mammalian sperm physiology and fertilizing capacity (Gopalakrishnan et al., 1998; Aydos et al., 2009; Safarinejad et al., 2010; Vani et al., 2010; Tang et al., 2012; Kan et al., 2013; Lakpour et al., 2013; Song et al., 2013; Kolesnikova et al., 2017; Llavanera et al., 2019b, 2020). On the other hand, a recent study established JNK signaling cascade as a regulator of specific physiological parameters in sperm cells (Luna et al., 2017). In this regard, under physiological conditions, GSTP1 is a well-known regulator of the JNK singling pathway in somatic cells by inhibiting its kinase activity when forming a GSTP1–JNK heterocomplex (Adler et al., 1999; Turella et al., 2005). However, the effects of inhibiting GSTP1 upon JNK signaling regulation in male gametes have never been investigated. To the best of our knowledge, this is the first report uncovering the physiological role of JNK inhibited by GSTP1 in mammalian sperm physiology. To this end, a specific inhibitor of the GSTP1–JNK heterocomplex was used, leading to the subsequent activation of JNK.

While the presence of GSTP1 in the sperm was established by proteomic studies in several mammalian species such as human (Wang et al., 2013), murine (Vicens et al., 2017), porcine (Pérez-Patino et al., 2019), and bovine (Peddinti et al., 2008), its localization was determined for the first time in the present study. Immunoblotting analysis of the present study identified a single ∼48 kDa band corresponding to GSTP1. Although the molecular mass of GSTP1 is ∼24 kDa, it is known to exist intracellularly as homodimers (Okamura et al., 2015), which is likely to be responsible for the ∼48 kDa band found in immunoblots. Furthermore, immunofluorescence analysis found GSTP1 to be localized in the posterior region of the head and the middle, principal, and end pieces of the tail in fresh control samples. The localization pattern of GSTP1 in fresh samples was similar to that found for other GST family members such as GSTM3 in pig (Llavanera et al., 2020) and buffalo (Kumar et al., 2014) sperm, which is present in the entire sperm tail. Interestingly, liquid storage for 72 h rather than inhibition with TER was responsible for the alteration of the GSTP1 localization pattern. Contrary to fresh samples, GSTP1 was found to be localized in the equatorial subdomain of the head and the principal and end pieces of the tail. A similar modulation of the GSTP1 localization pattern due to liquid storage was observed in other GST family members such as GSTM3 (Llavanera et al., 2020). Contrary to that, GSTM3 was found to be relocalized to the middle piece during sperm cryopreservation (Llavanera et al., 2019a). According to the results of the present study, previous studies hypothesized that the GST localization pattern in the sperm tail and their relocalization from or to the middle piece during liquid storage or cryopreservation, respectively, could contribute to the explanation of their significant role in mitochondrial function, sperm motility, and membrane stability. Sperm GSTs are known to be membrane-anchored proteins, and thus, their localization is determined by membrane stability (Llavanera et al., 2019b). The loss of GSTP1 from the middle piece suggests stronger membrane destabilization of this region. In this regard, the loss of GSTP1 in the middle piece would indicate a major membrane destabilization of this region due to preservation in liquid storage.

As has been previously reported in the literature, the JNK activation is regulated by GSTP1 in somatic cells (Adler et al., 1999; Wang et al., 2001). However, there were no studies regarding this molecular interaction in mammalian sperm cells. Immunoblotting analysis of phospho-JNK reported herein showed an intensified tyrosine and threonine phosphorylation of this protein in TER-treated samples, a specific blocker of the JNK-binding site in GSTP1. It is widely known that mitogen-activated protein kinases (MAPKs; e.g., JNKs) are activated via a dual phosphorylation upon tyrosine and threonine residues (Lawler et al., 1998). Hence, our results evidence, for the first time in mammalian sperm, the role of the GSTP1–JNK heterocomplex as an inhibitor of JNK activation by preventing the dual phosphorylation of tyrosine and threonine residues.

An interesting physiological effect of the activation of JNK was the significant decrease in sperm mitochondrial activity, viability, and motility. Activation of JNK has been reported in the literature to be related to mitochondrial dysfunction and cell death in somatic cells (Aoki et al., 2002; Heslop et al., 2020). Admittedly, a study conducted in ram sperm (Luna et al., 2017) showed that phosphorylation of sperm JNK increased apoptotic-like changes and DNA damage as well as capacitation-related events. These results would suggest that the GSTP1–JNK heterocomplex could prevent sperm to undergo early capacitation-related events or apoptotic-like changes during liquid storage. In this regard, the detrimental effects of JNK activation upon mitochondrial functionality in sperm cells showed herein are in agreement with the results reported in sperm and other cell types. Moreover, the reduction of mitochondrial activity is likely to be responsible for the loss of sperm motility, since mammalian sperm rely upon high levels of the adenosine triphosphate (ATP) required for axonemal dynein to drive sperm motility (Vívenes et al., 2009). Altogether, our findings suggest the role of the GSTP1–JNK heterocomplex in preserving sperm mitochondrial activity and subsequent viability and motility as well as in preventing capacitation-related events or apoptotic-like changes. Specific molecular mechanisms through which JNK activation may trigger sperm mitochondrial dysfunction in sperm cells remains to be determined. However, in somatic cells, JNK-mitochondrial SH3-domain binding protein 5 (SAB), a docking protein for JNK, has been suggested as a putative responsible for these processes, since it was found to lead to an intramitochondrial signal transduction pathway that impairs mitochondrial activity and enhances the production of reactive oxygen species (Win et al., 2018). In this regard, further investigations on the downstream effects of activated JNK upon mitochondrial activity should be performed.

Related to sperm mitochondrial dysfunction, the results of the present study showed an increase in intracellular superoxide levels triggered by the GSTP1–JNK heterocomplex dissociation and subsequent activation of JNK. Similar results were reported in somatic cells, where JNK activation was related to increased superoxide formation (Heslop et al., 2020). The main superoxide source in mammalian sperm cells is known to be the mitochondria, specifically, the electron transport chain (Storey, 2008; Brand, 2016). These results suggest that, in line with the previously mentioned results, the activation of JNK would lead to the disruption of the electron transport chain of sperm mitochondria. Moreover, previous studies in caprine and porcine evidenced the essential role of sperm GSTs in maintaining mitochondrial activity and physiological levels of reactive oxygen species (Hemachand and Shaha, 2003; Llavanera et al., 2020). Related with this, the results of the present study would indicate that the effects of GSTs upon sperm mitochondria would be mediated by a JNK signaling pathway. However, further research regarding the molecular mechanism of GSTs in regulating sperm mitochondrial function is required.

Our results showed that pharmacological dissociation of the GSTP1–JNK heterocomplex in sperm cells significantly impaired the stability of lipidic membranes, although it did not affect the acrosome membrane. Previous studies utilizing general GST inhibitors in goat and pig sperm reported high levels of plasma membrane damage and destabilization, although they did not find any effect on the acrosomal membrane (Gopalakrishnan and Shaha, 1998; Llavanera et al., 2020). These evidences reveal a significant role of these antioxidant enzymes on the stability of sperm plasma membrane but not on that of acrosome membrane. In accordance with the previously reported results, these findings could suggest a specific destabilization of the membranes located in the middle and principal pieces rather than from the sperm head, which could cause mitochondrial and motility impairment. However, the specific localization and molecular mechanisms by which GSTs are able to maintain membrane stability are currently unknown. The results of the present study shed some light on the mechanisms regulating destabilization of sperm membranes, suggesting that this process could be mediated by the activation of JNK signaling. However, the specific JNK downstream signaling proteins are yet to be determined. Uncovering the specific molecular signaling pathway through which sperm membrane stability is reduced is of utmost interest to develop new strategies for increasing sperm life span and quality.

Interestingly, although GSTP1–JNK dissociation caused severe mitochondrial damage and membrane destabilization in sperm cells, it did not have any effects upon intracellular calcium reservoirs. In this sense, previous studies in pig sperm showed that general GST inhibitors caused a significant increase in calcium levels, predominantly in the sperm middle piece (Llavanera et al., 2020). The present results suggested that, despite some specific GST classes being involved in the regulation of sperm calcium levels, the inhibition of GSTP1 upon JNK seems not to be related to calcium fluctuations. However, further research tackling calcium levels due to JNK activation should be performed in order to confirm this hypothesis.

**FIGURE 11:**
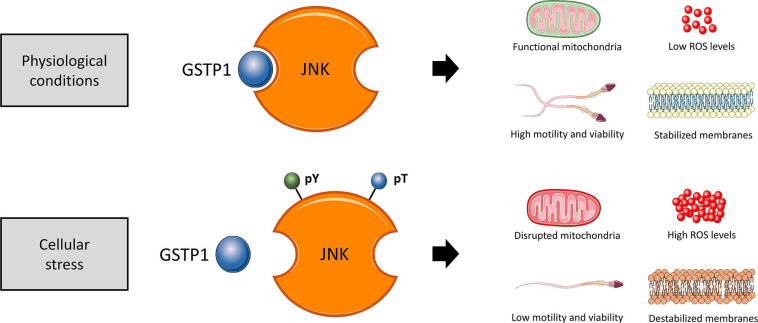
Proposed diagram of the function of the GSTP1–JNK heterocomplex under physiological conditions and cellular stress in sperm cells. In physiological conditions, the GSTP1–JNK heterocomplex prevents JNK activation, maintaining sperm viability, motility, mitochondrial activity, and plasma membrane stability and increased superoxide levels. On the other hand, under cellular stress conditions, the GSTP1–JNK heterocomplex is dissociated, and thus, JNK is activated by tyrosine (pY) and threonine (pT) phosphorylation. The activation of JNK decreases sperm viability and motility, disrupts mitochondrial activity, causes plasma membrane destabilization, and increases intracellular superoxide levels.

In conclusion, immunological and cell biology analyses confirmed that, as schematized in Figure 11, the dissociation of the GSTP1–JNK heterocomplex results in the activation of JNK and significantly declines sperm viability, motility, mitochondrial activity, and plasma membrane stability and increased superoxide levels, without altering intracellular calcium levels and acrosome membrane integrity. Thus, the present study provides several evidences supporting the molecular role of JNK activation via dissociation of the GSTP1–JNK heterocomplex, uncovering the role of this protein in maintaining sperm functionality, especially with regard to the preservation of mitochondrial physiology. These findings set the grounds for understanding the relevance of GSTP1–JNK cell signaling regulation in mammalian sperm physiology.

## Data Availability Statement

The original contributions presented in the study are included in the article/Supplementary Material, further inquiries can be directed to the corresponding authors.

## Author Contributions

MY and ML: conceptualization and methodology. ML, SO, YM-O, AD-B, and SR: formal analysis and investigation. ML: writing-original draft preparation. MY and IB: writing-review and editing and supervision. MY: funding acquisition. All authors contributed to the article and approved the submitted version.

## Conflict of Interest

The authors declare that the research was conducted in the absence of any commercial or financial relationships that could be construed as a potential conflict of interest.

## Supplementary Material

Supplementary Figure 1 Preliminary concentration test. Mean and standard error of the mean (SEM) of the percentage of **(A)** viable sperm, **(B)** total motile sperm, **(C)** viable-membrane destabilized sperm, and **(D)** high mitochondrial membrane potential (high MMP) sperm. Different ezatiostat (TER) concentrations (10 μM, 100 μM, and 1000 μM) were tested (TER 72h; light gray bars), and the same volume of DMSO was added to Control-72h samples as a vehicle control group (Control 72h; medium gray bars). Semen samples at 0 h of storage were also analyzed (Control 0h; dark gray bars). Sample size (*n* = 3). ^∗^*p* ≤ 0.05; ^∗∗^*p* ≤ 0.01. ^∗∗∗^*p* ≤ 0.001. A concentration of 10 μM of TER showed no effect on any sperm quality parameter compared to Control 0h and 72h samples (*p* > 0.05). Sperm samples treated with 100 μM showed a significant decrease in total sperm motility (*p* < 0.001) and MMP (*p* < 0.01), an increased sperm membrane destabilization (*p* < 0.001), and a minor but significant decrease in sperm viability (*p* < 0.05), compared to the Control 0h and 72h samples. Finally, samples treated with 1000 μM of TER showed significant (*p* < 0.001) detrimental effects upon all sperm quality parameters, especially on sperm viability, compared to the Control 0h and 72h samples. These results suggested that samples treated with 10 μM of TER did not exert any effect upon sperm physiology, whereas 1000 μM of TER caused cytotoxic effects on sperm and thus masking the physiological effects of the inhibitor upon sperm quality and functionality. Finally, samples treated with 100 μM of TER showed physiological effects upon sperm membranes, mitochondria, and motility without exerting critical cytotoxicity.

Supplementary Table 1 Raw data. Raw data of sperm quality and functionality parameters of all treatments and time points. TMOT, percentage of total motile sperm; PMOT, percentage of progressively motile sperm; VAP, sperm average path velocity (μm/s); VIABILITY, percentage of viable sperm (SYBR-14^+^/PI^–^); M540, percentage of membrane-destabilized cells (M540^+^) within the total viable sperm population (Yo-Pro-1^–^); HE, relative fluorescence intensity of viable sperm with high levels of intracellular O_2_^–⋅^ (E^+^/PI^–^); JC1, percentage of high mitochondrial membrane potential sperm (ΔΨm) resulted from the orange-stained (JC1_*agg*_) population; PNA, percentage of acrosome membrane-intact sperm (PNA-FITC^–^) within the total viable sperm population (PI^–^); FL3, relative Fluo3-AM fluorescence intensity of viable sperm with high levels of intracellular calcium (Fluo3-AM^+^/PI^–^); CNT 0h, semen samples at 0 h of storage; CNT 72h, vehicle control (dimethyl sulfoxide; DMSO) semen samples at 72 h of storage at 17°C; TER 72h, semen samples treated with 100 μM ezatiostat (TER) for 72 h of storage at 17°C.
