# Supplementary figures and images for: Deactivation of the JNK Pathway by GSTP1 Is Essential to Maintain Sperm Functionality
Source: Front Cell Dev Biol. 2021 Feb 25;9:627140. doi: 10.3389/fcell.2021.627140 (PMC7959831; doi:10.3389/fcell.2021.627140)

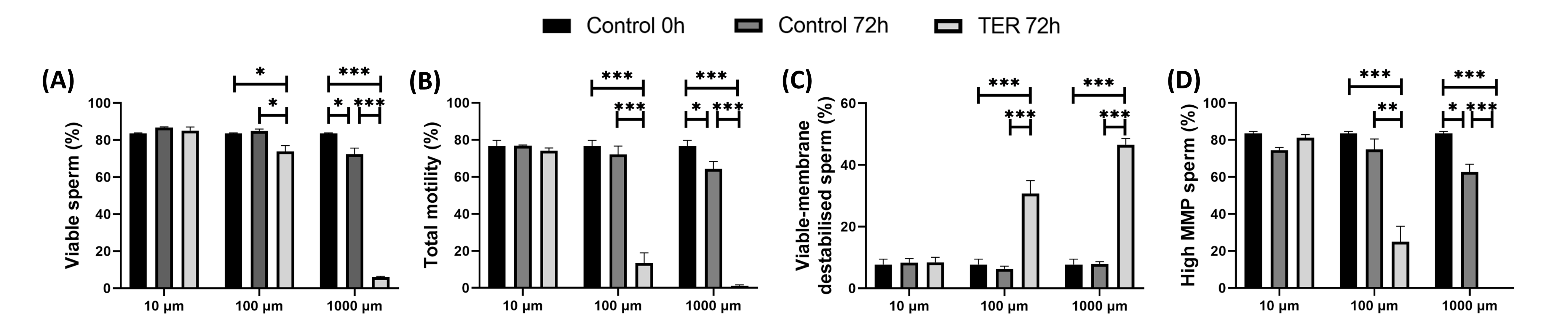

Supplement: Supplementary Figure 1 — Preliminary concentration test. Mean and standard error of the mean (SEM) of the percentage of (A) viable sperm, (B) total motile sperm, (C) viable-membrane destabilized sperm, and (D) high mitochondrial membrane potential (high MMP) sperm. Different ezatiostat (TER) concentrations (10 μM, 100 μM, and 1000 μM) were tested (TER 72h; light gray bars), and the same volume of DMSO was added to Control-72h samples as a vehicle control group (Control 72h; medium gray bars). Semen samples at 0 h of storage were also analyzed (Control 0h; dark gray bars). Sample size (n = 3). ∗p ≤ 0.05; ∗∗p ≤ 0.01. ∗∗∗p ≤ 0.001. A concentration of 10 μM of TER showed no effect on any sperm quality parameter compared to Control 0h and 72h samples (p > 0.05). Sperm samples treated with 100 μM showed a significant decrease in total sperm motility (p < 0.001) and MMP (p < 0.01), an increased sperm membrane destabilization (p < 0.001), and a minor but significant decrease in sperm viability (p < 0.05), compared to the Control 0h and 72h samples. Finally, samples treated with 1000 μM of TER showed significant (p < 0.001) detrimental effects upon all sperm quality parameters, especially on sperm viability, compared to the Control 0h and 72h samples. These results suggested that samples treated with 10 μM of TER did not exert any effect upon sperm physiology, whereas 1000 μM of TER caused cytotoxic effects on sperm and thus masking the physiological effects of the inhibitor upon sperm quality and functionality. Finally, samples treated with 100 μM of TER showed physiological effects upon sperm membranes, mitochondria, and motility without exerting critical cytotoxicity. [file Image_1.TIF]
