# Supplementary material for: Deactivation of the JNK Pathway by GSTP1 Is Essential to Maintain Sperm Functionality
Source: Front Cell Dev Biol. 2021 Feb 25;9:627140. doi: 10.3389/fcell.2021.627140 (PMC7959831; doi:10.3389/fcell.2021.627140)
Supplement: Supplementary File 1 — Supplementary information for Materials and Methods. [file Table_2.DOCX]

Supplementary Material

# Supplementary information for Materials and Methods

**Flow cytometry analyses**

*General information*

Sperm viability (SYBR-14/propidium iodide; SYBR-14/PI), membrane stability (merocyanine 540/Yo-Pro-1; M540/Yo-Pro-1), mitochondrial activity (5,5′,6,6′-tetrachloro-1,1′,3,3′tetraethyl-benzimidazolylcarbocyanine iodide; JC1), intracellular calcium levels (Fluo3-AM/propidium iodide; Fluo3/PI), intracellular superoxide levels (hydroethidine/Yo-Pro-1; HE/Yo-Pro-1) and acrosome membrane integrity (fluorescein-conjugated peanut agglutinin/propidium iodide; PNA/PI) were evaluated in every treatment and time.

Samples were diluted with pre-warmed PBS to a final concentration of 2×10^6^ cells per mL in a final volume of 0.6 mL before they were stained with the corresponding protocol. The flow cytometry assessments were conducted using a Cell Laboratory QuantaSC cytometer (Beckman Coulter; Fullerton, CA, USA), and samples were excited with an argon ion laser (488 nm) set at a power of 22 mW. Laser voltage and rate was constant along the experiment. The cytometer provided the electronic volume (EV) and the side scatter (SS) for each event. Three optical filters (FL-1, FL-2 and FL-3) were used. FL-1 served to detect green fluorescence (SYBR-14, Yo-Pro-1, 5,5’,6,6’-tetrachloro-1,1’,3,3’tetraethyl-benzimidazolylcarbocyanine iodide monomers (JC-1 monomers; JC-1_mon_), peanut agglutinin conjugated with fluorescein isothiocyanate (PNA-FITC) and Fluo-3-AM), FL-2 was used to detect orange fluorescence (JC-1 aggregates; JC-1_agg_) and FL-3 allowed to detect red fluorescence (merocyanine 540 (M540), hydroethidine (HE) and propidium iodide (PI)). Signals were logarithmically amplified, and photomultiplier settings were adjusted to particular staining methods.

Unstained and single-stained samples for each fluorochromes were used for setting the electronic volume (EV) gain, FL-1, FL-2 and FL-3 PMT-voltages and for compensating spill over other channels. Sperm population was distinguished from debris using the EV. A total of three technical replicates, with 10,000 events per replicate, were evaluated for each sample and sperm parameter. Flowing Software (Ver. 2.5.1; University of Turku, Finland) was used to perform flow cytometric data analysis, following the recommendations of the International Society for Advancement of Cytometry (ISAC). The corresponding mean ± standard error of the mean (SEM) was subsequently calculated.

*Sperm viability (SYBR-14/PI)*

Sperm viability was evaluated by assessing their membrane integrity using the LIVE/DEAD sperm viability kit (Molecular Probes, Eugene, OR, USA), which follows the protocol of Garner and Johnson [1]. Briefly, spermatozoa were stained with SYBR-14 (final concentration: 100 nmol/L) for 10 min at 38ºC in the dark and with PI (final concentration: 12 µmol/L) for 5 min at the same conditions. Combination of SYBR-14 and PI resulted in three sperm populations in flow cytometry dot-plots: (i) viable green-stained spermatozoa (SYBR-14^+^/PI^-^); (ii) non-viable red-stained spermatozoa (SYBR-14^-^/PI^+^); and (iii) moribund spermatozoa stained both green and red (SYBR-14^+^/PI^+^). Non-sperm particles (debris; SYBR-14^-^/PI^-^) appeared in the left-bottom quadrant. Viable green-stained spermatozoa (SYBR-14^+^/PI^-^) were used to assess sperm viability. SYBR-14 spill over into the FL3-channel was compensated (2.45%). The percentage of particles within the double negative (left bottom) quadrant of every parameter described above was corrected using the debris particles found in SYBR-14^-^/PI^-^ population. The percentages of the other three populations were also recalculated. Finally, the corrected percentage of SYBR-14^+^/PI^-^ sperm was used to evaluate sperm viability.

*Sperm membrane lipid disorder (M540/YO-PRO-1)*

Membrane lipid disorder of sperm was evaluated by M540 and Yo-Pro-1 co-staining, following the procedure of Rathi et al. [2] with minor modifications by Yeste et al. 2014 [3]. Briefly, spermatozoa were incubated with M540 (final concentration: 2.6 µmol/L) and Yo-Pro-1 (final concentration: 25 nmol/L) for 10 min at 38ºC in the dark. M540 is a hydrophobic fluorochrome which is able to intercalate into the membrane. As membrane fluidity increases M540 uptake, this fluorochrome is stablished a marker for destabilisation of sperm plasma membrane validated in boar, bull, stallion and dog sperm [4]. The combination of both fluorochromes resulted in four populations: (i) viable spermatozoa with low membrane lipid disorder (M540^-^/Yo-Pro-1^-^); (ii) viable spermatozoa with high membrane lipid disorder (M540^+^/Yo-Pro-1^-^); (iii) non-viable spermatozoa with low membrane lipid disorder (M540^-^/Yo-Pro-1^+^) and (iv) non-viable spermatozoa with high membrane lipid disorder (M540^+^/Yo-Pro-1^+^). Data were not compensated. Data was not compensated. The percentage of membrane destabilised cells (M540^+^) within the total viable sperm population (Yo-Pro-1^-^) was used to assess membrane lipid disorder.

*Mitochondrial membrane potential (JC-1)*

ΔΨm of sperm was evaluated following a modified protocol from Ortega-Ferrusola et al. [5]. All samples were incubated with JC-1 (final concentration: 0.3 µmol/L) for 30 min at 38ºC in the dark. High ΔΨm causes JC-1 aggregates that emit orange fluorescence collected through FL-2, whereas low ΔΨm causes JC-1 monomers that emit green fluorescence collected through FL-1. Consequently, a total of three sperm populations were observed in flow cytometry dot-plots: (i) spermatozoa with low ΔΨm (green-stained); (ii) spermatozoa with high ΔΨm (orange-stained) and (iii) spermatozoa with heterogeneous mitochondria (green and orange-stained in the same cell). Data was not compensated. Spermatozoa considered having high ΔΨm (JC1_agg_), resulted from the orange-stained populations, were used to assess ΔΨm.

*Intracellular calcium levels (Fluo3-AM/PI)*

Midpiece and head intracellular calcium of spermatozoa was determined using the modified protocol described by Harrison et al. [6]. Samples were incubated for 10 min at 38ºC with Fluo3-AM (final concentration: 1 µmol/L) and PI (final concentration: 12 µmol/L). Fluo3-AM is a non-fluorescent non-polarized membrane-permeable dye that, upon binding to calcium, emit green fluorescence [7]. Previous studies in boar sperm showed that Fluo3-AM marks both head and midpiece calcium deposits [8]. Accordingly, four sperm populations were identified in dot plots: i) viable spermatozoa with low levels of intracellular calcium (Fluo3-AM^-^/PI^-^); ii) viable spermatozoa with high levels of intracellular calcium (Fluo3-AM^+^/PI^-^); iii) non-viable spermatozoa with low levels of intracellular calcium (Fluo3-AM^-^/PI^+^), and iv) non-viable spermatozoa with high levels of intracellular calcium (Fluo3-AM^+^/PI^+^). Compensation of Fluo3-AM spill over into FL-3 (2.45%) and PI spill over into FL-1 (28.72%) was performed. The relative Fluo3-AM fluorescence intensity of viable sperm with high levels of intracellular calcium (Fluo3-AM^+^/PI^-^) was used to assess sperm head and midpiece calcium levels.

*Total intracellular O_2_^-^● levels (HE/YO-PRO-1)*

Total sperm intracellular superoxide (O_2_^-^●) levels were evaluated following a modification of the procedure described by Guthrie and Welch [9]. Sperm samples were incubated with HE (final concentration of 4 µmol/L) and with YO-PRO-1 (final concentration of 40 nmol/L), at 38°C and for 20 min in the dark. The oxidation of HE to ethidium (E^+^) by O_2_^-^● was detected as red fluorescence through FL-3 and green fluorescence from YO-PRO-1 was detected through FL-1. Combination of these fluorochromes resulted in four populations: (i) non-viable spermatozoa with high O_2_^-^● levels (E^++^/YO-PRO-1^+^), (ii) viable spermatozoa with high O_2_^-^● levels (E^++^/YO-PRO-1^-^), (iii) non-viable spermatozoa with low O_2_^-^● levels (E^+-^/YO-PRO-1^+^); and (iv) viable spermatozoa with low O_2_^-^● levels cells (E^+-^/YO-PRO-1^-^). YO-PRO-1 spill over into the FL3-channel was compensated (5.06%). Spermatozoa considered having high O_2_^-^● levels (E^++^/YO-PRO-1^-^) were used to asses O_2_^-^● levels. The relative HE fluorescence intensity of viable sperm with high levels of intracellular O_2_^-^● (E^+^/PI^-^) was used to assess sperm intracellular superoxide levels.

*Acrosome membrane integrity analysis (PNA-FITC/PI)*

Sperm acrosome membrane intactness was evaluated according to the modified procedure described by Nagy et al. [10]. Sperm samples were incubated with PNA-FITC (final concentration: 2.5 µg/mL) for 5 min at 38 ºC in the dark. Subsequently, cells were stained with PI (final concentration: 12 µmol/L) for 5 min at 38ºC. PNA-FITC is a hydrophobic enzyme that binds to the outer acrosomal membrane of sperm [11]. As spermatozoa were not previously permeabilised, the four following sperm populations were distinguished: i) viable membrane-intact spermatozoa (PNA-FITC^-^/PI^-^); ii) non-viable spermatozoa with damaged plasma membrane that presented an outer acrosome membrane that could not be fully intact (PNA-FITC^+^/PI^+^); iii) non-viable spermatozoa with damaged plasma membrane and fully-lost outer acrosome membrane (PNA-FITC^-^/PI^+^); and iv) viable spermatozoa with damaged plasma membrane (PNA-FITC^+^/PI^-^). Compensation of PNA-FITC spill over into the PI channel (2.45%) was performed. The percentage of acrosome membrane-intact sperm (PNA-FITC^-^) within the total viable sperm population (PI^-^) was used to assess acrosome membrane integrity.

**References cited in Supplementary Information for Materials and Methods**

1. Garner, D.L.; Johnson, L.A. Viability assessment of mammalian sperm using SYBR-14 and propidium iodide. *Biol. Reprod.* **1995**, *53*, 276–84, doi:10.1095/biolreprod53.2.276.

2. Rathi, R.; Colenbrander, B.; Bevers, M.M.; Gadella, B.M. Evaluation of in vitro capacitation of stallion spermatozoa. *Biol. Reprod.* **2001**, *65*, 462–70, doi:10.1095/biolreprod65.2.462.

3. Yeste, M.; Estrada, E.; Rivera del Álamo, M.-M.; Bonet, S.; Rigau, T.; Rodríguez-Gil, J.-E. The increase in phosphorylation levels of serine residues of Ppotein HSP70 during holding time at 17°C is concomitant with a higher cryotolerance of boar spermatozoa. *PLoS One* **2014**, *9*, e90887, doi:10.1371/journal.pone.0090887.

4. Steckler, D.; Stout, T.A.E.; Durandt, C.; Nöthling, J.O. Validation of merocyanine 540 staining as a technique for assessing capacitation-related membrane destabilization of fresh dog sperm. *Theriogenology* **2015**, *83*, 1451–1460, doi:10.1016/j.theriogenology.2015.01.019.

5. Ortega-Ferrusola, C.; Sotillo-Galan, Y.; Varela-Fernandez, E.; Gallardo-Bolanos, J.M.; Muriel, A.; Gonzalez-Fernandez, L.; Tapia, J.A.; Pena, F.J. Detection of “apoptosis‐like” changes during the cryopreservation process in equine sperm. *J. Androl.* **2007**, *29*, 213–221, doi:10.2164/jandrol.107.003640.

6. Harrison, R.A.P.; Mairet, B.; Miller, N.G.A. Flow cytometric studies of bicarbonate‐mediated Ca2+ influx in boar sperm populations. *Mol. Reprod. Dev.* **1993**, *35*, 197–208, doi:10.1002/mrd.1080350214.

7. Takahashi, A.; Camacho, P.; Lechleiter, J.D.; Herman, B. Measurement of Intracellular Calcium. *Physiol. Rev.* **1999**, *79*, 1089–1125, doi:10.1152/physrev.1999.79.4.1089.

8. Yeste, M.; Fernández-Novell, J.M.; Ramió-Lluch, L.; Estrada, E.; Rocha, L.G.; Cebrián-Pérez, J.A.; Muiño-Blanco, T.; Concha, I.I.; Ramírez, A.; Rodríguez-Gil, J.E. Intracellular calcium movements of boar spermatozoa during “in vitro” capacitation and subsequent acrosome exocytosis follow a multiple-storage place, extracellular calcium-dependent model. *Andrology* **2015**, *3*, 729–747, doi:10.1111/andr.12054.

9. Guthrie, H.D.; Welch, G.R. Determination of intracellular reactive oxygen species and high mitochondrial membrane potential in Percoll-treated viable boar sperm using fluorescence-activated flow cytometry. *J. Anim. Sci.* **2006**, *84*, 2089–2100, doi:10.2527/jas.2005-766.

10. Nagy, S.; Jansen, J.; Topper, E.K.; Gadella, B.M. A Triple-Stain Flow Cytometric Method to Assess Plasma- and Acrosome-Membrane Integrity of Cryopreserved Bovine Sperm Immediately after Thawing in Presence of Egg-Yolk Particles. *Biol. Reprod.* **2003**, *68*, 1828–1835, doi:10.1095/biolreprod.102.011445.

11. Mortimer, D.; Curtis, E.F.; Miller, R.G. Specific labelling by peanut agglutinin of the outer acrosomal membrane of the human spermatozoon. *J. Reprod. Fertil.* **1987**, *81*, 127–135, doi:10.1530/jrf.0.0810127.
